# Nephroprotective effects of *Feijoa Sellowiana* leaves extract on renal injury induced by acute dose of ecstasy (MDMA) in mice

**Published:** 2014-01

**Authors:** Mohammad Karami, Faezeh Karimian Nokabadi, Mohammad Ali Ebrahimzadeh, Farshad Naghshvar

**Affiliations:** 1 Department of Toxicopharmacology, School of Pharmacy, Mazandaran University of Medical Sciences, Sari, Iran; 2 Department of Chemistry, School of Pharmacy, Mazandaran University of Medical Sciences, Sari, Iran; 3 Department of Pathology, School of Medicine, Mazandaran University of Medical Sciences, Sari, Iran

**Keywords:** Ecstasy, *Feijoa sellowiana*, Kidney glutathione, MDMA, Nephrotoxicity

## Abstract

***Objective(s):*** Because of the high total phenolic contents of *Feijoa sellowiana*, its nephroprotective effect was determined in 3,4-methylene dioxymethamphetamine (MDMA) treated mice.

***Materials and Methods:*** Animals received 10, 20 and 40 mg/kg doses of aqueous or methanol extract of *F. sellowiana* leaves, intra-peritoneally. After one hour, an acute administration of MDMA (20 mg/kg, IP) was injected. Nephroprotective effect was assayed by determination of serum creatinine, serum urea and kidney glutathione level. Histopathological study was also used.

***Results*** **:** Both extracts at 40 mg/kg resulted in a significant reversal in the raised serum creatinine levels (*P* <0.05). Not statistically significant was observed in effect of two extracts (*P*>0.05). A decrease in urea/ creatinine ratio was observed following aqueous extract treatment. Methanolic extract showed higher activity in increasing kidney glutathione (*P*<0.001 compared to MDMA group). Methanol extract showed higher protective activity in histopathology study.

***Conclusion:*** *F. sellowiana* extract resulted in a markedly decrease in the nephrotoxicity of MDMA in mice.

## Introduction

*Feijoa sellowiana* (Myrtaceae) is an evergreen bush which is widely spread in the southern part of Iran. Due to its easy adaptability in subtropical regions, nowadays it is being extensively cultivated in many countries and also in Iran, where its fruit is widely popular. Although the chemical composition of *F. sellowiana* has been clearly reported, pharmacological studies of its constituents have barely been done (1). It showed potent antimicrobial and antifungal activity and a sensible activity against *Helicobacter pylori *(2, 3). Also anti-cancer activities of the full *F. sellowiana* extract have been reported (3). Limited information is available concerning application of *F. sellowiana* antioxidant activity (4). Moreover, antioxidant activities of aqueous extract on oxidative burst of human whole blood phagocytes have been reported (5). 3,4-methylenedioxymethamphetamine (MDMA, ecstasy) is a ring-substituted amphetamine derivative. It has also attracted a great deal of media attention in recent years due to its widespread abuse as a recreational drug by young people (6). Clinical evidence has shown that the kidney is a target for MDMA toxicity. In this sense, MDMA is metabolized by cytochromes P_450_ 2D, 2B and 3A and reactive metabolites are readily oxidized to the corresponding o-qiuinones and to the reactive oxygen species (6). Recently, hepatoprotective activity of *F. sellowiana *against MDMA-induced liver injury in mice has been reported by our group (7). Because of the high antioxidant activity of *F. sellowiana *and its high phenolic and flavonoids contents (4), nephroprotective effect of aqueous and methanolic extracts of *F. sellowiana *leaves was examined against MDMA treated mice.

## Materials and Methods

***Animals***

Male albino mice weighing 25-30 g were used for all experiments. They were housed in standard mice cages in a room with a 12 hr light-dark cycle at 22 ± 2C, and free access to food and water. The animals were adapted to the condition 7 days prior to the beginning of the experiment. The experiments were performed during the day time (08:00-16:00 hr). Each animal was used only once. A research proposal was prepared according to the guidelines of the Committee for the Purpose of Control and Supervision of Experiments on Animals (CPCSEA). The Institutional Animal Ethics Committee (IAEC) of Mazandaran University of Medical Sciences, Mazandaran, Iran also approved the proposal. 

**Table 1:** Biochemical analyses of the kidney glutathione and serum samples treated by aqueous and methanolic extracts of *Feijoa sellowiana* leaves

Groups	Dose (mg/ kg, IP)	Kidney glutathione (µmol/g)	Serum creatinine (mg/dl)	Serum urea (mg/dl)	Urea / Creatinine ratio
Control group	-	22.08	1.17	20.04	17.13
MDMA treated	20	6.46^c^	2.09^c^	49.19 ^c^	23.53^c^
Aqueous extract	10	6.70^ c^	2.03^ cns^	40.13^ cf^	19.77^be^
Aqueous extract	20	9.50^ ce^	1.98^cns^	37.86^ cf^	19.12^af^
Aqueous extract	40	13.08^ cf^	1.78^bd^	32.22^ cf^	18.10^nsf^
Methanolic extract	10	5.03^ c^	2.05^ cns^	44.50^ cf^	21.70^ bd^
Methanolic extract	20	14.39^ cf^	2.02^ cns^	42.74^ cf^	21.16^bd^
Methanolic extract	40	18.87^ af^	1.69^ bd^	36.85^ cf^	21.80^bd^

a, b and c represent significance at *P*<0.05, *P*<0.01 and *P*<0.001 levels, respectively, when compared to normal saline control (10 ml/ kg, IP) d, e and f represent significance at *P*<0.05, *P*<0.01 and *P*<0.001 levels, respectively when compared to MDMA treated group, ns represents non significance when compared to normal saline and MDMA treated control groups

***Extract preparation***

*F. sellowiana* leaves were collected from Fajr Citrus Experimental Institute in autumn, 2011. Leaves were dried at room temperature and coarsely grounded. Powder was extracted at room temperature by percolation with methanol (400 ml, 3 times) or water (400 ml, 3 times), separately. The resulting extracts were concentrated using a rotary vacuum until a crude solid extract was obtained. Extracts were dispersed in normal saline as vehicle for pharmacological studies. 

***Experimental design***

Animals were divided in eight groups each one consisted of five male mice. Normal saline (10 ml/kg, IP) was administered to group I, which served as negative control or control group (8).While group II was injected with MDMA (20 mg/ kg, IP) which served as MDMA group. The remaining six groups (10. Aqueous; 20. Aqueous; 40. Aqueous) and (10. Methanolic; 20. Methanolic; 40. Methanolic) were injected with 10, 20 and 40 mg/kg IP of either aqueous or methanolic extract of leaves, respectively. After one hour, animals received an acute dose of MDMA (20 mg/kg, IP) dispersed in normal saline. 

***Biochemical analyses***

Animals were fasted overnight but distilled water was available *ad libitum*. Twenty-four hours post-induction, mice were sacrificed using inhaled diethyl ether anaesthesia. Blood samples, obtained directly from the heart chamber of the anaesthetized mice, were left for complete clotting for about 5 hr before they were centrifuged with Uniscope Laboratory Centrifuge (Model SM 112, Surgifriend Medicals, England) at 3000 rpm for 20 min in order to separate the serum. The serum was carefully separated into new, well labeled, corresponding plain sample bottles at room temperature Serum urea and creatinine were estimated by Berthelot method (Urease enzymatic method) and Jaffe’s reaction, respectively, on standard diagnostic test kits of Zist Chimie (Tehran, Iran). Reduced glutathione was estimated according to the method described by Ellman et al (9). The yellow color developed was measured at 412 nm (UV- Visible EZ201, Perkin Elmer: USA). 

***Histopathological studies***

Mice were sacrificed and their kidneys were removed for histopathological examination. The kidneys were completely excised and any extraneous tissue was omitted. Multiple samples were then taken from each kidney (mean size: 3 mm) and placed in 10% neutral buffered formalin. It was then processed into 4-5 μm thick sections stained with hematoxylin-eosin and observed under a photomicroscope (Model N - 400ME, CEL-TECH Diagnostics, Hamburg, Germany). 

***Statistical analysis***

Statistical analysis was performed using SPSS for Windows (Ver.10, SPSS, Inc., Chicago, USA). Data were analyzed by one-way analysis of variance (ANOVA) followed by Tukey multiple comparison post test and expressed as mean ± SD. *P*< 0.05 was considered to be significant. 

## Results

***Serum urea***

All data are presented in Table 1. As compared to control group, a significant increase in serum urea level was observed in MDMA treated group (*P* <0.001, Table 1). 10. aqueous and 20. aqueous groups showed a significant dose depended decrease in blood urea levels as compared to MDMA treated group (*P*<0.001). Aqueous extract showed higher activity than methanolic extract in lowering serum urea concentration but this effect was not statistically significant (*P* >0.05). 

**Figure 1:**
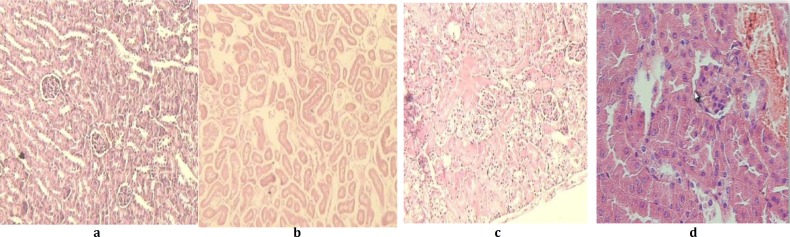
Photomicrograph of lobules from control, MDMA groups and 20. aqueous extract of extract* sellowiana* leaves treated kidney. Left is a representative section of a normal kidney (a), Second, an acute 20 mg/kg, IP of MDMA treated mice kidney (b) Third is a single dose of 20 mg/kg of aqueous extract of leaves, one hour before MDMA injection (c) and the fourth is a single dose of 20 mg/kg of methanolic extract of leaves, one hour before MDMA injection (d) Hematixylin and eosin (X25)

***Serum creatinine***

All data are presented in Table 1. MDMA treated group showed a significant increase in serum creatinine level when compared to control group (*P*< 0.001, Table 1). It was shown that, 40. aqueous and 40. methanolic groups resulted in a significant reversal in the raised serum creatinine levels when compared to MDMA treated group (*P*<0.05, Table 1). Other groups did not show any statistically significant activity as compared to MDMA treated group (*P*>0.05). The effect was dose dependent but only the highest dose in each groups showed statistically significant activity.

***Urea / Creatinine ratio***

Serum urea / serum creatinine ratio which is a more useful indicator, was also calculated and presented in Table 1. As compared to control group, a significant increase in this ratio was observed in MDMA treated group (*P*<0.001). It was recorded that, 10. Aqueous group, and 20. Aqueous groups showed a significant decrease in urea / creatinine ratio as compared to MDMA treated group, respectively. Also, 40. Aqueous extract group was completely effective as It reversed this ratio to normal value (18.10 *vs*. 17.13, *P*>0.05). Methanolic extracts showed a weak activity in lowering this ratio (*P*<0.05, as compared to MDMA group). 

***Kidney glutathione***

As shown in Table 1, MDMA treated group showed a significant decrease in glutathione level when compared to control group (*P*<0.001). Extracts showed a significant and dose depended increase in kidney glutathione as compared to MDMA treated group. Methanolic extract groups showed higher activity in increasing kidney glutathione. Moreover**, **40. Methanolic extract as the most potent extract, increased glutathione level from 6.46 (MDMA treated group) to 18.87 µmol/g (*P*< 0.001, as compared to MDMA group). Normal value was 22.08 µmol/g (*P*< 0.05, as compared to control group).

***Light microscope observation***

Light microscope histopathological studies showed significantly decrease in the kidney cellular damage, including necrosis; hemorrhage and edema by aqueous extract treated groups (Figure 1.c) as compared to MDMA treated and control groups (Figures 1a and 1b). 

## Discussion

MDMA is believed to be the primary toxic constituent that is present in ecstasy. Other toxic constituents have been also identified including MDA (3, 4 methylenedioxy amphetamine) and DOM (4-methyl-2,5-dimethoxy amphetamine). MDMA was also shown to be an inhibitor of glutathione peroxidase, which catalyzed the destruction of H_2_O_2_ of lipid hydroperoxidase by reduced glutathione. Therefore, with inhibition of glutathione peroxidase, there is a reduction in GSH which results in an accelerated lipid peroxidation (10, 11). Antioxidants such as vitamin E and selenium have been proposed to prevent membrane damage of lipid peroxidation not only through glutathione peroxidase but also by allowing hydrogen to be abstracted from their own structure rather than from the allylic hydrogen of on unsaturated lipid, thus interrupting with the free radical chain reaction (12). Treatment with aqueous extract of *F. sellowiana* leaves showed a significant decrease in the toxicity of MDMA in treated mice (Figure 1c). Accumulation of *Feijoa* extract components in the kidney as the target organ for toxicity, may be a reason for this protection (13). Our study improved renal protective activity (Table 1). This could be a result of *F. sellowiana* extract receptor binding, which is sufficient to affect different cells (14). This may be through the mechanism mentioned above as well as good reductive capability of extract to reduce Fe^+3^ to Fe^+2^ by donating electrons, Fe^+2^ chelating activity and / or anti-lipid peroxidation activities (15). Further investigation on individual compounds, in parallel with their *in vivo* antioxidant activities and assessment of different antioxidant mechanisms seem necessary. 

## Conclusion

Aqueous extract of *F. sellowiana* leaves showed a significant decrease in MDMA-induced nephrotoxicity in mice. The antioxidant activity of *F. sellowiana* may be a possible mechanism.
